# The interrater reliability of static palpation of the thoracic spine for eliciting tenderness and stiffness to test for a manipulable lesion

**DOI:** 10.1186/s12998-018-0218-7

**Published:** 2018-12-04

**Authors:** Amber M. Beynon, Jeffrey J. Hebert, Bruce F. Walker

**Affiliations:** 1 School of Health Professions, Murdoch University, 90 South Street, Murdoch, WA 6150 Australia; 2 Faculty of Kinesiology, University of New Brunswick, 3 Bailey Drive, Fredericton, NB E3B 5A3 Canada; 3 School of Psychology and Exercise Science, Murdoch University, 90 South Street, Murdoch, 6150 WA Australia

**Keywords:** “Static palpation”, Thoracic, Reliability, Chiropractic, Tenderness, “Manipulable lesion”

## Abstract

**Background:**

Despite widespread use by manual therapists, there is little evidence regarding the reliability of thoracic spine static palpation to test for a manipulable lesion using stiffness or tenderness as diagnostic markers. We aimed to determine the interrater agreement of thoracic spine static palpation for segmental tenderness and stiffness and determine the effect of standardised training for examiners. The secondary aim was to explore expert consensus on the level of segmental tenderness required to locate a “manipulable lesion”.

**Methods:**

Two experienced chiropractors used static palpation of thoracic vertebrae on two occasions (pragmatic and standardised approaches). Participants rated tenderness on an 11-point numerical pain rating scale (NPRS) and raters judged segmental stiffness based on their experience and perception of normal mobility with the requested outcomes of hypomobile or normal mobility. We calculated interrater agreement using percent agreement, Cohen’s Kappa coefficients (***κ***) and prevalence-adjusted bias-adjusted Kappa coefficients (PABAK). In a preliminary study, an expert panel of 10 chiropractors took part in a Delphi process to identify the level of meaningful segmental tenderness required to locate a “manipulable lesion”.

**Results:**

Thirty-six participants (20 female) were enrolled for the reliability study on the 13th March 2017. Mean (SD) age was 22.4 (3.4) years with an equal distribution of asymptomatic (*n* = 17) and symptomatic (n = 17) participants. Overall, the interrater agreement for spinal segmental stiffness had Kappa values indicating less than chance agreement [***κ*** range − 0.11, 0.53]. When adjusted for prevalence and bias, the PABAK ranged from slight to substantial agreement [0.12–0.76] with moderate or substantial agreement demonstrated at the majority of spinal levels (T1, T2 and T6 to T12). Generally, there was fair to substantial agreement for segmental tenderness [Kappa range 0.22–0.77]. Training did not significantly improve interrater agreement for stiffness or tenderness. The Delphi process indicated that an NPRS score of 2 out of 10 identified a potential “manipulable lesion”.

**Conclusion:**

Static palpation was overall moderately reliable for the identification of segmental thoracic spine stiffness and tenderness, with tenderness demonstrating a higher reliability. Also, an increased agreement was found within the mid-thoracic spine. A brief training intervention failed to improve reliability.

**Electronic supplementary material:**

The online version of this article (10.1186/s12998-018-0218-7) contains supplementary material, which is available to authorized users.

## Background

“Manipulable lesion” is a diagnostic term often used by manual therapists [1]. Static palpation is commonly used by chiropractors and manual therapists to detect manipulable lesions [2]. In essence, it is used clinically to assess areas of pain and stiffness within the spine that, may indicate spinal dysfunction and identify the location of treatment for manual therapists [3]. Spinal manipulation is a manual treatment where a vertebral joint is passively moved using a low-amplitude high-velocity thrust [4]. The appropriate application of spinal manipulation to areas of “spinal dysfunction” is thought to improve segmental function and motion, with reductions in pain and associated symptoms [5]. Manipulable lesions identified by static palpation have been described as increased stiffness, a decrease in the segmental joint and musculature elasticity or springiness, and increased tenderness [6–9].

Bergmann and Peterson define static palpation as “Palpation of bony landmarks (that) incorporates a scanning assessment of contour, tenderness, and alignment of the spinous processes, transverse processes, rib angles, interspinous space and intercostal space.” [10]. According to this definition of static palpation one element is to determine tenderness; but how much tenderness is needed? There has been no previous agreement on the magnitude of tenderness needed to determine whether a manipulable lesion is present or not. This question could be answered through a consensus of experts using a method such as the Delphi technique.

Studies that examined static palpation of the thoracic spine, have shown inconsistent evidence with fair (κ: 0.24) [11] to substainal (κ: 0.67) [12] agreement for tenderness but only slight agreement with (κ: 0.07) [6] to (κ: 0.15) [11] for stiffness. Studies evaluating the reliability of static palpation in the cervical and lumbar spine have also shown mixed results ranging from poor [8] to almost perfect [13] (κ range 0.03 to 0.90 respectively). Generally, there is a low reliability with static palpation alone, however in combination with palpation of tender segments, a higher reliability level has been reported [14–17].

Questions remain regarding the reliability of static palpation. Previous research has focussed on palpation of the cervical and lumbar spine, although chiropractors commonly treat the thoracic spine [13]. There is little evidence on the reliability of static palpation to test for manipulable lesion through tenderness and stiffness within the thoracic spine. It would therefore be beneficial for patient diagnosis and potentially patient outcome to determine if static palpation of the thoracic spine is a reliable measure. Also, there are no known studies that have determined an agreement on the magnitude of tenderness needed to determine whether a manipulable lesion is notionally present or not.

The primary aims of this study were to establish the interrater reliability of static palpation of the thoracic spine for eliciting tenderness and segmental spinal stiffness and determine the effect of standardised training [10] for examiners on these outcomes. The secondary aim was to explore expert consensus on the level of segmental tenderness required to locate “manipulable lesion”.

## Methods

### Preliminary Delphi study

We surveyed an expert panel of chiropractors to answer the question “Using the numerical pain rating scale, what minimum level of quantifiable tenderness/pain should a person experience at a segmental spinal level to be regarded as a potential manipulable lesion?” The process was an iterative one exploring opinions with the aim of reaching an 80% consensus. We then used this tenderness level as the minimum needed to determine a manipulable lesion in the pain section of the reliability study. This research had ethics approval from Murdoch University Human Research Ethics Committee (2016/162), and all participants were provided written informed consent.

We recruited an expert panel of 10 chiropractors with more than 3 years’ clinical experience from the Murdoch University School of Health Professions. As per Delphi methodology we had the option of using three to four iterative phases [18]. The first started with the primary question with additional space for participants to add any pertinent comments. The second phase was designed to gain an understanding of how all involved participants viewed the feedback from the first round and the primary question was re-asked. In phase three we combined the pain scale ratings provided so that participants could give a final well-informed answer having considered peer responses [18]. Respondents remained anonymous.

During each phase of the Delphi method mean, standard deviations, range, mode and median were calculated using SPSS version 24 [19].

### Reliability study design

We used a repeated measures design. Two chiropractors examined participants on two occasions. First, the raters performed static palpation using their usual method to attempt to simulate their usual clinical practice (pragmatic approach), and then again after a training session using the method of static palpation as per Bergmann and Peterson [10]. We piloted the complete study procedure on four participants prior to full data collection and this did not result in any changes to our methods. The reliability study was approved by Murdoch University Human Research Ethics Committee (2016/160), and all participants gave informed consent.

We recruited adult participants with or without spinal pain from the Murdoch University student population. Exclusion criteria were based on previous studies [3, 12, 20] and comprised the following self-reported diagnosis of: fibromyalgia, osteoarthritis, ankylosing spondylitis, rheumatoid arthritis, or other inflammatory spinal disease, previous back surgery, known scoliosis greater than 40 degrees, a history of recent severe thoracic spine trauma, unable to lay prone including pregnancy, or unable to tolerate physical examination due to severe back pain requiring medical attention or prescription drugs within the last 3 weeks. The Stata v14.2 sskdlg module was used for a power analysis. A sample size of 32 participants was needed using the parameters of a kappa of 0.5, the proportion of first and second observer to rate positives of 0.1 and an absolute precision of 0.5 with a 95% level of confidence. Four more participants were recruited than the power analysis calculation to account for the possibility of a 5% drop out on the day.

Two experienced chiropractors with 3 years’ clinical experience acted as raters. A third examiner (anatomist), or a fourth examiner (chiropractor) used a non-toxic, non-permanent skin marking pen to mark the perceived levels of the spinous processes of thoracic vertebrae 1 to 12, to eliminate between-examiner error in identifying the nominated spinal level. Only one examiner marked each participant’s spinous processes.

The first stage involved pragmatic static palpation. Participants were randomly assigned to rater 1 or 2 in different rooms. They then crossed over to the other respective rater. Upon completion, the raters participated in a 45-min training session that included verbal and written explanations of Bergmann and Peterson’s [10] standardized method of static palpation. To ensure that the raters correctly understood the standardized approach, their knowledge of the method was evaluated with a written examination. After successful training, there was a second data collection stage using the standardized method of static palpation. The same participants were again randomly assigned to rater 1 or 2 to reduce memory bias of the pragmatic round. Participants then crossed over to the other respective rater. Each rater was blinded to the other’s findings, and both raters and participants were blinded to their own previous examination findings. This was achieved using an additional person who recorded the results given by the examiners through non-verbal cues.

### Palpation process

Static palpation involved a “spring test” performed centrally over the spinous processes of the vertebrae to assess segmental mobility and pain provocation. It was defined as a gentle but firm, anteriorly directed pressure [8]. While the participants were in a prone position raters palpated each of the thoracic spinal levels from 1 to 12. Raters examined for the presence of segmental stiffness. The interpretation of whether a segment was stiff or not was in relation to the segments above and below and is a subjective estimate based on the examiner’s experience and perception of normal flexibility. They then gained feedback from the participant on whether the segment was tender or not and rated this on an 11-point numeric pain rating scale (NPRS), with the explanation that scores of 0 represented no pain and 10 the worst imaginable pain.

During the training session the raters received a verbal and written explanation of Bergmann and Peterson’s (2010) standardized method of static palpation, which is as follows;

*“Static palpation of the spine and posterior chest wall is commonly performed with the patient in the prone position. During the evaluation, stand to the side of the patient and accommodate to the patient by bending at the knees, hips and waist. Palpation typically begins with an assessment of superficial temperature and sensitivity, followed by the assessment of consistency and mobility of the dermal layer and muscular layer. Palpation of bony landmarks incorporates a scanning assessment of contour, tenderness, and alignment of the spinous processes, transverse processes, rib angles, interspinous space and intercostal space. Tenderness and alignment of the spinous processes, interspinous spaces, and transverse processes are assessed with unilateral or bilateral fingertip contacts.”*

### Statistical analysis

Percent agreement, Cohen’s Kappa coefficients, proportion of maximum possible Kappa (Kappa max) and Prevalence-Adjusted Bias-Adjusted Kappas (PABAK) with 95% confidence intervals were calculated with SPSS version 24 [19] and DAG_Stat [21]. The standard error of PABAK was calculated as 4Po(1-Po)/N, where Po = observed proportion of agreement [Po = (a + d)/N], N = total number of cases and transformed to 95% confidence intervals with the following formula: Kpb ± 1.96√(SE of Kpb), where Kpb = PABAK and SE = standard error [22–24].

Percent agreement does not take into account chance agreement. We interpreted Kappa values using the Landis and Koch [25] classification scale where below chance agreement < 0.00, slight agreement 0.00–0.20, fair agreement 0.21–0.40, moderate agreement 0.41–0.60, substantial agreement 0.61–0.80, and almost perfect agreement 0.81–1.00. Kappa can be adjusted to account for prevalence and bias. Prevalence occurs when the proportion on the positive classification differs from the negative classification. If the prevalence of a yes is either very low or very high, the chance agreement is also high and therefore Kappa is reduced. Bias occurs when the raters disagree on the proportion of yes and no judgments. As bias increases, chance agreement would decrease, therefore inflating the Kappa estimate [22, 26]. Kappa max acts to measure the strength of agreement while maintaining the proportions of positive rating demonstrated by each rater, it demonstrates the degree to which the raters’ ability to agree is constrained by pre-existing factors [26].

The interrater agreement of strict agreement between the raters (agreement with respect to a single vertebral segment on the same side eg. left T8) were calculated for the pragmatic method and standardized method, for stiffness and tenderness. Additionally, the adjacent vertebral levels were then expanded (three levels combined into one) and interrater agreement was recalculated across the expanded levels. Expanding levels and recalculating scores was based on an assumption that palpatory pressure on one segment may impact on the level above and below, clinically an “area” of symptoms is usually treated with spinal manipulative therapy rather than a specific vertebral segment [12]. Forest plots were created to show a visual representation of PABAK values. We used a Kappa cut-off of 0.60 to indicate meaningful reliability based on McHugh [27].

## Results

### Preliminary study of pain threshold

There was a response rate of 80%. At the end of phase three of the Delphi study, a 75% consensus was reached from the 8 expert participants who completed all three surveys. Consensus identified a minimum reported segmental tenderness of 2 out of 10 on the NPRS to be considered as a potential manipulable lesion.

Throughout the phases of the Delphi study the standard deviation and range decreased and the mean, median and mode became closer to 2. In phase 3, 75% of responders scored 2 out of 10 as the minimal tenderness level needed. It was decided to end the Delphi study at Phase 3 and accept the 75% consensus rate as the frequency statistics were convincingly indicating 2 out of 10 as the minimal measurement to indicate a potential manipulable lesion.

### Reliability

On the 13th March 2017 a total of 36 participants were enrolled in the reliability study. Of these, 34 participants (20 female) completed data collection and were included in the analysis; two participants failed to complete the second round of data collection owing to time constraints. Average age was 22.44 (S.D. 3.41, range 18–37 years) with an even distribution of both asymptomatic (*n* = 17) and symptomatic (*n* = 17) participants. Two experienced chiropractors with 3 years’ clinical experience acted as raters.

The interexaminer reliability for strict agreement of the pragmatic approach for spinal stiffness, ranged from Kappa of − 0.31 to 0.47 depending on the spinal level. An additional file shows full results (see Additional file 1). Regarding the interexaminer reliability for strict agreement of the standardized approach to segmental stiffness, Kappa ranged from − 0.11 to 0.53. When Kappa was adjusted for prevalence and bias (PABAK) there was an increase agreement level in both the pragmatic approach and standardized approach ranging from − 0.14 to 0.71 and 0.12 to 0.76 respectively. An additional file shows full results (see Additional file 2).

The interexaminer reliability for the strict agreement of the pragmatic approach to segmental tenderness, Kappa ranged from 0.22 to 0.77. An additional file shows full results (see Additional file 3). The interexaminer reliability for strict agreement of the standardized approach to segmental tenderness, Kappa ranged from 0.25 to 0.70. An additional file shows full results (see Additional file 4). The standardized method did not increase reliability markedly in determining segmental tenderness for strict agreement. When assessing tenderness Kappa was adjusted for prevalence and bias (PABAK) with the level of agreement remaining similar in both rounds.

**Fig. 1:**
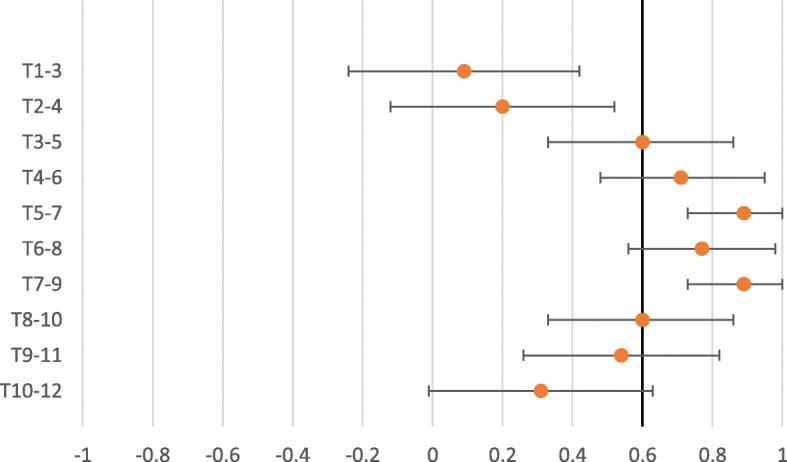
Expanded vertebra- PABAK with 95% confidence intervals. Pragmatic approach to assess segmental stiffness

**Fig. 2:**
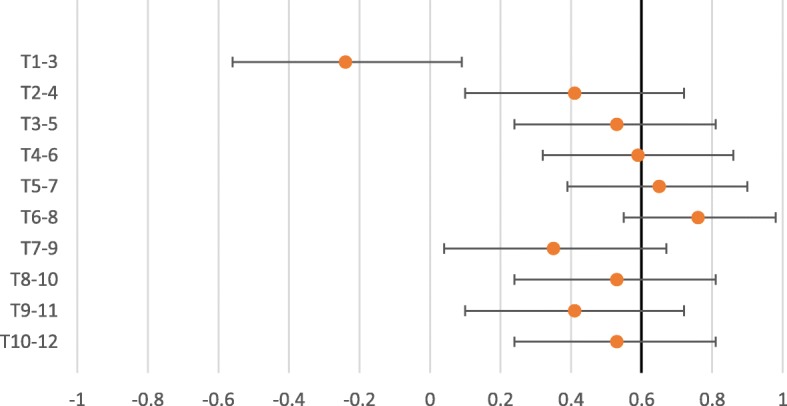
Expanded vertebra- PABAK with 95% confidence intervals. Standardized approach to assess segmental stiffness

The interexaminer reliability for expanded vertebra agreement for the pragmatic approach to segmental stiffness, Kappa ranged from − 0.25 to 0.30 (Table 1). The interexaminer reliability for expanded vertebra agreement for the standardized approach to segmental stiffness shows Kappa ranged from − 0.11 to 0.30 (Table 2). The PABAK values ranged from 0.09 to 0.89 in the pragmatic approach (Table 1) and − 0.24 to 0.76 in the standardised approach (Table 2). As seen in the forest plots for the pragmatic approach to segmental stiffness (Fig. 1), of the PABAK reliability scores, six out of 10 were above the cut-off line (0.60) and for the standardized approach to segmental stiffness (Fig. 2), two out of 10 were above the cut-off line (0.60) to demonstrate meaningful reliability [27]. However, it should be noted that the confidence intervals of all but two of the PABAK reliability scores for segmental stiffness intersect with the cut-off line. The Kappa max values ranged from − 0.25 to 0.46 in the pragmatic approach (Table 1) and − 0.69 to 0.46 in the standardised approach (Table 2). One hundred percent of the Kappa max values fall within the 95% confidence intervals of the Kappa score or the PABAK results.

**Table 1:** Interexaminer reliability- Expanded vertebra agreement- Pragmatic approach to assess segmental stiffness

Spinal level	Kappa	95%CI	PABAK	95% CI	Indicates	Kappa max
T1–3	− 0.20	− 0.46, 0.07	0.09	− 0.24, 0.42	Slight agreement	−0.19
T2–4	−0.25	− 0.38, − 0.12	0.20	−0.12, 0.52	Slight agreement	−0.25
T3–5	0.12	−0.26, 0.50	0.60	0.33, 0.86	Moderate agreement	0.20
T4–6	−0.07	−0.14, 0.00	0.71	0.48, 0.95	Substantial agreement	−0.09
T5–7	−0.01	−0.01, 0.00	0.89	0.73, 1.00	Almost perfect agreement	−0.01
T6–8	−0.06	−0.12, 0.00	0.77	0.56, 0.98	Substantial agreement	−0.06
T7–9	−0.03	−0.07, 0.01	0.89	0.73, 1.00	Almost perfect agreement	−0.03
T8–10	−0.05	−0.14, 0.04	0.60	0.33, 0.86	Moderate agreement	−0.20
T9–11	0.30	−0.06, 0.66	0.54	0.26, 0.82	Moderate agreement	0.46
T10–12	0.05	−0.28, 0.37	0.31	−0.01, 0.63	Fair agreement	0.07

**Table 2:** Interexaminer reliability- Expanded vertebra agreement- Standardized approach to assess segmental stiffness

Spinal level	Kappa	95%CI	PABAK	95% CI	Indicates	Kappa max
T1–3	−0.13	−0.34, 0.08	− 0.24	−0.56, 0.09	Below chance agreement	−0.33
T2–4	0.04	−0.25, 0.33	0.41	0.10, 0.72	Moderate agreement	0.09
T3–5	−0.05	−0.15, 0.04	0.53	0.24, 0.81	Moderate agreement	−0.14
T4–6	−0.11	−0.20, − 0.03	0.59	0.32, 0.86	Moderate agreement	−0.13
T5–7	−0.10	− 0.17, − 0.02	0.65	0.39, 0.90	Substantial agreement	− 0.09
T6–8	− 0.06	−0.12, 0.00	0.76	0.55, 0.98	Substantial agreement	−0.06
T7–9	0.06	−0.29, 0.41	0.35	0.04, 0.67	Fair agreement	0.07
T8–10	0.30	−0.07, 0.66	0.53	0.24, 0.81	Moderate agreement	0.46
T9–11	−0.10	−0.23, 0.02	0.41	0.10, 0.72	Moderate agreement	−0.22
T10–12	−0.05	−0.15, 0.04	0.53	0.24, 0.81	Moderate agreement	−0.14

**Fig. 3:**
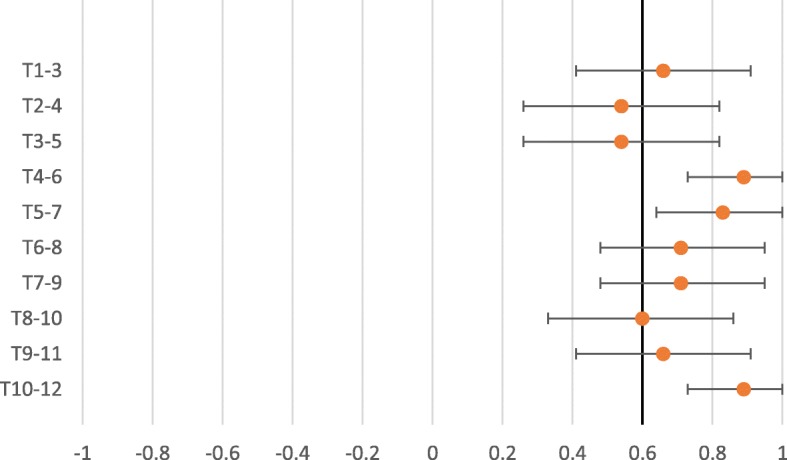
Expanded vertebra- PABAK with 95% confidence intervals. Pragmatic approach to assess segmental tenderness

**Fig. 4:**
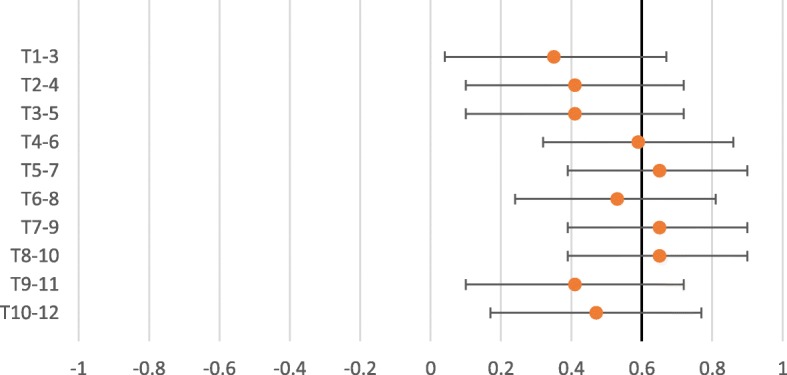
Expanded vertebra- PABAK with 95% confidence intervals Standardized approach to assess segmental tenderness

The interexaminer reliability for expanded vertebra agreement for the pragmatic approach to segmental tenderness, Kappa values ranged from 0.23 to 0.85 (Table 3). The interexaminer reliability for expanded vertebra agreement for the standardized approach to segmental tenderness, showed Kappa values ranging from 0.18 to 0.56 (Table 4). The PABAK ranged from 0.54 to 0.89 in the pragmatic approach (Table 3) and 0.35 to 0.65 in the standardized approach (Table 4) demonstrating an increase as compared to the Kappa. As seen in the forest plot for the pragmatic approach to segmental tenderness (Fig. 3), of the PABAK reliability score, eight out of 10 were above the cut-off line (0.60) and in the standardized approach to segmental tenderness (Fig. 4), three out of 10 were above the cut-off line (0.60) demonstrating meaningful reliability [27]. However, it should be noted that the confidence intervals of all but three of the PABAK reliability scores for segmental tenderness intersect with the cut-off line. The Kappa max values ranged from 0.35 to 1.00 in the pragmatic approach (Table 3) and 0.18 to 1.00 in the standardised approach (Table 4). Eighty-five percent of the Kappa max values fall within the 95% confidence intervals of the Kappa score or the PABAK results.

**Table 3:** Interexaminer reliability- Expanded vertebra agreement- Pragmatic approach to assess segmental tenderness

Spinal level	Kappa	95%CI	PABAK	95% CI	Indicates	Kappa max
T1–3	0.41	0.04, 0.78	0.66	0.41, 0.91	Substantial agreement	0.67
T2–4	0.23	−0.11, 0.58	0.54	0.26, 0.82	Moderate agreement	0.55
T3–5	0.21	−0.16, 0.58	0.54	0.26, 0.82	Moderate agreement	0.35
T4–6	0.64	0.19, 1.00	0.89	0.73, 1.00	Almost perfect agreement	1.00
T5–7	0.52	0.06, 0.99	0.83	0.64, 1.00	Almost perfect agreement	0.62
T6–8	0.36	−0.08, 0.81	0.71	0.48, 0.95	Substantial agreement	0.42
T7–9	0.46	0.06, 0.86	0.71	0.48, 0.95	Substantial agreement	0.52
T8–10	0.41	0.04, 0.77	0.60	0.33, 0.86	Moderate agreement	0.44
T9–11	0.56	0.26, 0.86	0.66	0.41, 0.91	Substantial agreement	0.79
T10–12	0.85	0.65, 1.00	0.89	0.73, 1.00	Almost perfect agreement	1.00

**Table 4:** Interexaminer reliability- Expanded vertebra agreement- Standardized approach to assess segmental tenderness

Spinal level	Kappa	95%CI	PABAK	95% CI	Indicates	Kappa max
T1–3	0.26	−0.06, 0.58	0.35	0.04, 0.67	Fair agreement	0.39
T2–4	0.25	−0.10, 0.60	0.41	0.10, 0.72	Moderate agreement	0.29
T3–5	0.18	−0.18, 0.55	0.41	0.10, 0.72	Moderate agreement	0.18
T4–6	0.41	0.05, 0.76	0.59	0.32, 0.86	Moderate agreement	0.55
T5–7	0.46	0.10, 0.82	0.65	0.39, 0.90	Substantial agreement	0.56
T6–8	0.36	0.01, 0.70	0.53	0.24, 0.81	Moderate agreement	0.53
T7–9	0.52	0.20, 0.84	0.65	0.39, 0.90	Substantial agreement	0.76
T8–10	0.56	0.28, 0.85	0.65	0.39, 0.90	Substantial agreement	1.00
T9–11	0.41	0.13, 0.70	0.41	0.10, 0.72	Moderate agreement	1.00
T10–12	0.48	0.22, 0.75	0.47	0.17, 0.77	Moderate agreement	0.80

## Discussion

Overall reliability of static palpation for segmental tenderness showed a higher level of reliability than palpation for stiffness which is in accord with previous literature [14, 15]. There is a higher level of reliability of static palpation within the mid-thoracic spine when assessing for tenderness.

The Delphi Study showed a minimum of 2 out of 10 on the NPRS was required to be a potential manipulable lesion suggesting that tenderness should not just be a yes/no question. In a study of this nature, it seems preferable to use the NPRS and a potential manipulable lesion is scored as a NPRS score above 2 out of 10. This finding should assist with any potential limitation that pain and tenderness are subjective measurements.

There was a good range of participants with a mix of female and male participants, and equal numbers of asymptomatic and symptomatic participants. This mix is consistent with recommendations to assemble a study sample better matching clinical practice [15, 28]. The mean age of participants at 22.4 years is younger than many of the previous studies [12, 29] however was similar to some other studies [6, 11, 30].

The low level of reliability for determining segmental stiffness for strict agreement could be due to the high level of possible chance agreement. This gave high prevalence indices which led to an underestimation of the Kappa coefficient. We used a prevalence-adjusted, bias-adjusted kappa (PABAK), and this accounted for the high prevalence bias. When this bias was adjusted for, there was moderate reliability for most spinal levels. This is also reflected in the Kappa max values. Our results have produced higher levels of agreement when compared to other studies, however most previous studies did not account for prevalence bias. When Schneider and others [20] did account for potential prevalence bias they also found a higher level of reliability.

In comparing other reliability studies of static palpation, the findings are similar to our own i.e. a low level of reliability with static palpation alone for spinal stiffness. Ghoukassian, Nicholls and McLaughlin [6] examined interexaminer reliability using the Johnston and Friedman method for thoracic spine palpation, they found slight interexaminer reliability, Kappa of 0.07 [6]. Potter, McCarthy, and Oldham [31] examined the intraexaminer reliability of multiple examination procedures including range of motion, motion palpation, and static palpation and found an intraclass correlation coefficient in the thoracic spine of 0.70 (95%[CI], 0.27–0.90). Cooperstein, Haneline, and Young [32] considered the examiners’ confidence of their judgements and assessed the most ‘fixated’ level of the thoracic spine, they found overall a poor intraclass correlation coefficient (0.31), however when both examiners were “very confident” in their findings, analysis of this subgroup population (40% of participants) showed an increase in the intraclass correlation coefficient to 0.83 (95% [CI], 0.63–0.92).

There was a slight increase in the reliability of static palpation of spinal stiffness with training, however this was not statistically significant (*p* = 0.39). Interesting there was a higher level of reliability of static palpation within the mid-thoracic spine compared to the upper and lower thoracic spine when assessing for stiffness. We speculate that the anatomy of the thoracic spine in the mid region may be easier to palpate given its flexibility to anterior forces in a prone position.

When comparing our findings on static palpation for tenderness of the thoracic spine we found similar results to Christensen and others [12], where their population age was similar to ours. They examined palpation tenderness of thoracic vertebral levels 1–8, and found with an expanded agreement an intraexaminer reliability Kappa of 0.59 to 0.77 and an interexaminer reliability Kappa of 0.67 to 0.70. Johnston and others [33] examined the interexaminer reliability of paraspinal soft tissue tension by percussion finding 70–86% overall agreement. Dissimilar to our findings, Heiderscheit and Boissonnault [11], examined static palpation with pain provocation in a population with ages similar to ours, and found pain provocation intraexaminer reliability with a Kappa of 0.28 to 0.66 and interexaminer reliability with a Kappa of 0.24.

We did find that reliability moderately increased with expanded vertebra for spinal segmental tenderness and for segmental stiffness, and this is understandable as collapsing levels for analysis delivers an inherently increased potential for agreement.

Overall there was a relatively low level of reliability for static palpation when testing for stiffness, and a higher level of reliability found for static palpation when testing for tenderness. Segmental assessment for stiffness is not sufficiently reliable, but improves when considering a region (multi-levels of vertebrae). Therefore, in clinical practice chiropractors may need only be concerned with approximate levels and any more detailed analysis using static palpation could be of limited utility. Also, reliability is better in the mid-thoracic spine when compared with the lower and upper thoracic spine which has direct clinical implications for spinal assessment. There was no significant difference in reliability for spinal stiffness and tenderness after a training session suggesting that the pragmatic approaches used by two experienced chiropractors were equivalent.

### Strengths of study

The strengths of this study were it was fully powered, we blinded participants and examiners, we used randomization before each round and attempted to follow best practice recommendations from the literature. We carried out a training session with a consensus method [9, 34] as per Bergmann and Peterson [10] and marked thoracic spinous processes [5, 34]. We explored the reliability of pain provocation assessment [11, 15, 34] and rated this level of tenderness [35]. Also during data analysis we not only calculated Kappa but also PABAK [20], and analysed strict agreement and expanded agreement [12].

### Limitations of study

A limitation of the Delphi study was that there was only a 75% consensus. Although we did not reach the ideal 80% consensus the frequency statistics were overwhelmingly indicating 2 out of 10 on the NPRS. Within the Delphi study we defined an expert as having over 3 years’ clinical experience however we cannot guarantee that a similar sample of chiropractors would necessarily generate similar results. All expert chiropractors were recruited from Murdoch University School of Health Professions, however, they graduated from many different institutions worldwide. Nevertheless, recruiting from the one institution may limit external validity. The examiner training for standardization appeared adequate given the knowledge assessment however the training was brief and additional training may have additionally enhanced the reliability. Further, as the difference between the pragmatic and standardized approaches were not significant it could indicate that the training was inadequate or possibly the examiners were trained in this or a similar method during their studies. The large number of statistical comparisons may have increased the probability of type I error. Another limitation is the use of a non-clinical population, while there was a mixture of symptomatic and asymptomatic participants for spinal pain the participants were mostly healthy young students. This may adversely affect external validity. The examiners reported that towards the end they were experiencing fatigue and this may have influenced the results leading to a lower level of agreement.

## Conclusion

A Delphi study of 10 experienced chiropractors concluded that the minimum level of quantifiable tenderness at a segmental spinal level should be 2 out of 10 on the NPRS to be considered a potential manipulable lesion. There was no significant impact on reliability with standardized training for stiffness or tenderness. There is a higher level of reliability of static palpation within the mid-thoracic spine when assessing both stiffness and tenderness. There was overall moderate reliability for static palpation for stiffness and tenderness, with tenderness showing a higher level of reliability. Reliability modestly increased when three adjacent vertebral levels were expanded for analysis, both for spinal segmental stiffness and tenderness. These reliability results should be taken into consideration in clinical practice when assessing the spine particularly as the validity of static palpation is still unknown.

Future research could consider static palpation reliability in different patient groups such as those with overt patient syndromes. Additionally, when assessing the reliability of segmental stiffness, PABAK is beneficial as issues related to prevalence or bias can result in a lower level of perceived reliability.

## Additional files

Additional file 1: Interexaminer reliability- Strict agreement- Pragmatic approach to assess segmental stiffness. Table of results for strict agreement for the pragmatic approach to assess segmental stiffness. (PDF 201 kb)

Additional file 2: Interexaminer reliability- Strict agreement- Standardized approach to assess segmental stiffness. Table of results for strict agreement for the standardized approach to assess segmental stiffness. (PDF 200 kb)

Additional file 3: Interexaminer reliability- Strict agreement- Pragmatic approach to assess segmental tenderness. Table of results for strict agreement for the pragmatic approach to assess segmental tenderness. (PDF 199 kb)

Additional file 4: Interexaminer reliability- Strict agreement- Standardized approach to assess segmental tenderness. Table of results for strict agreement for the standardized approach to assess segmental tenderness. (PDF 200 kb)

## Abbreviations

CI: Confidence Intervals
ICC: Intraclass correlation coefficient
K: Kappa
Kappa max: proportion of maximum possible Kappa
N: Number of participants
NPRS: numerical pain rating scale
PABAK: prevalence-adjusted bias adjusted Kappa
T1: first thoracic vertebra (of twelve)
T1-T12: All thoracic vertebrae from the first to the twelfth inclusive
