# Supplementary material for: The interrater reliability of static palpation of the thoracic spine for eliciting tenderness and stiffness to test for a manipulable lesion
Source: Chiropr Man Therap. 2018 Dec 4;26:49. doi: 10.1186/s12998-018-0218-7 (PMC6278006; doi:10.1186/s12998-018-0218-7)
Supplement: Supplementary file 1 — Interexaminer reliability- Strict agreement- Pragmatic approach to assess segmental stiffness. Table of results for strict agreement for the pragmatic approach to assess segmental stiffness. (PDF 201 kb) [file 12998_2018_218_MOESM1_ESM.pdf]

Additional file 1: Interexaminer reliability- Strict agreement- Pragmatic approach to assess segmental stiffness

Spinal level	% Agreement	95% CI	Kappa	95%CI	PABAK	95% CI	Indicates	Kappa max
T1 Left	83	66, 93	0.18	-0.22, 0.58	0.66	0.41, 0.91	Substantial agreement	0.40
T2 Left	80	63, 92	-0.09	-0.18, 0.00	0.60	0.34, 0.86	Moderate agreement	-0.17
T3 Left	74	57, 88	-0.10	-0.21, 0.01	0.49	0.20, 0.77	Moderate agreement	-0.25
T4 Left	63	45, 79	-0.07	-0.35, 0.20	0.26	-0.06, 0.58	Fair agreement	-0.12
T5 Left	77	60, 90	0.20	-0.19, 0.59	0.54	0.26, 0.82	Moderate agreement	0.25
T6 Left	77	60, 90	-0.09	-0.20, 0.01	0.54	0.26, 0.82	Moderate agreement	-0.21
T7 Left	80	63, 92	-0.05	-0.14, 0.04	0.60	0.33, 0.86	Moderate agreement	-0.21
T8 Left	77	60, 90	-0.12	-0.21, -0.03	0.54	0.26, 0.82	Moderate agreement	-0.17
T9 Left	66	48, 81	0.05	-0.17, 0.27	0.31	-0.01, 0.63	Fair agreement	0.24
T10 Left	83	66, 93	-0.09	-0.17, -0.02	0.66	0.41, 0.91	Substantial agreement	-0.09
T11 Left	86	70, 95	-0.05	-0.13, 0.03	0.71	0.48, 0.95	Substantial agreement	-0.13
T12 Left	86	70, 95	-0.05	-0.13, 0.00	0.71	0.48, 0.95	Substantial agreement	-0.05
T1 Right	51	34, 69	-0.31	-0.46, 0.15	0.03	-0.30, 0.36	Slight agreement	-0.40
T2 Right	63	45, 79	0.11	-0.20, 0.41	0.26	-0.06, 0.58	Fair agreement	0.20
T3 Right	74	57, 88	0.03	-0.32, 0.38	0.49	0.20, 0.77	Moderate agreement	0.03
T4 Right	83	66, 93	0.47	0.11, 0.83	0.66	0.41, 0.91	Substantial agreement	0.57
T5 Right	63	45, 79	0.16	-0.18, 0.50	0.26	-0.06, 0.58	Fair agreement	0.17
T6 Right	57	39, 74	0.04	0.29, 0.36	0.14	-0.18, 0.47	Slight agreement	0.05
T7 Right	43	26, 61	-0.17	-0.49, 0.14	-0.14	-0.47, 0.18	Below chance agreement	-0.23
T8 Right	53	37, 71	0.05	-0.28, 0.38	0.09	-0.24, 0.42	Slight agreement	0.04
T9 Right	60	42, 76	0.02	-0.31, 0.36	0.20	-0.12, 0.52	Slight agreement	0.03
T10 Right	51	34, 69	-0.05	-0.35, 0.24	0.03	-0.30, 0.36	Slight agreement	-0.09
T11 Right	66	48, 81	-0.21	-0.32, -0.09	0.31	-0.01, 0.63	Fair agreement	-0.21
T12 Right	71	54, 85	-0.14	-0.25, -0.02	0.43	0.13, 0.73	Moderate agreement	-0.25
