# Supplementary material for: The interrater reliability of static palpation of the thoracic spine for eliciting tenderness and stiffness to test for a manipulable lesion
Source: Chiropr Man Therap. 2018 Dec 4;26:49. doi: 10.1186/s12998-018-0218-7 (PMC6278006; doi:10.1186/s12998-018-0218-7)
Supplement: Supplementary file 2 — Interexaminer reliability- Strict agreement- Standardized approach to assess segmental stiffness. Table of results for strict agreement for the standardized approach to assess segmental stiffness. (PDF 200 kb) [file 12998_2018_218_MOESM2_ESM.pdf]

Additional file 2: Interexaminer reliability- Strict agreement- Standardized approach to assess segmental stiffness

Spinal level	% Agreement	95% CI	Kappa	95%CI	PABAK	95% CI	Indicates	Kappa max
T1 Left	65	46, 80	-0.11	-0.24, 0.03	0.29	-0.03, 0.62	Fair agreement	-0.42
T2 Left	62	44, 78	0.01	-0.30, 0.32	0.23	-0.09, 0.56	Fair agreement	0.01
T3 Left	56	38, 73	-0.01	-0.01, 0.00	0.12	-0.22, 0.45	Slight agreement	-0.01
T4 Left	65	46, 80	0.04	-0.29, 0.36	0.29	-0.03, 0.61	Fair agreement	0.05
T5 Left	62	44, 78	-0.01	-0.33, 0.32	0.23	-0.09, 0.56	Fair agreement	-0.01
T6 Left	65	46, 80	0.05	-0.17, 0.27	0.29	-0.03, 0.61	Fair agreement	0.23
T7 Left	79	62, 91	0.24	-0.17, 0.65	0.59	0.32, 0.86	Moderate agreement	0.27
T8 Left	71	53, 85	-0.10	-0.23, 0.02	0.41	0.10, 0.72	Moderate agreement	-0.31
T9 Left	88	73, 97	-0.06	-0.12, 0.00	0.76	0.55, 0.98	Substantial agreement	-0.06
T10 Left	74	56, 87	-0.06	-0.15, 0.04	0.48	0.17, 0.77	Moderate agreement	-0.31
T11 Left	82	65, 93	0.21	-0.14, 0.56	0.65	0.39, 0.90	Substantial agreement	1.00
T12 Left	79	62, 91	-0.01	-0.01, 0.00	0.59	0.32, 0.86	Moderate agreement	-0.01
T1 Right	88	73, 97	-0.05	-0.12, 0.02	0.76	0.55, 0.98	Substantial agreement	-0.09
T2 Right	76	59, 89	0.07	-0.30, 0.43	0.53	0.24, 0.81	Moderate agreement	0.09
T3 Right	68	49, 83	-0.01	-0.01, 0.00	0.35	0.04, 0.67	Fair agreement	-0.01
T4 Right	65	46, 80	0.02	-0.31, 0.36	0.29	-0.03, 0.61	Fair agreement	0.03
T5 Right	65	46, 80	-0.06	-0.35, 0.23	0.29	-0.03, 0.61	Fair agreement	-0.09
T6 Right	71	53, 85	0.19	-0.17, 0.55	0.41	0.10, 0.72	Moderate agreement	0.23
T7 Right	68	49, 83	0.25	-0.09, 0.59	0.35	0.04, 0.67	Fair agreement	0.30
T8 Right	68	49, 83	-0.01	-0.30, 0.28	0.35	0.04, 0.67	Fair agreement	-0.02
T9 Right	79	62, 91	0.34	-0.05, 0.72	0.59	0.32, 0.86	Moderate agreement	0.37
T10 Right	62	44, 78	0.20	-0.13, 0.54	0.23	-0.09, 0.56	Fair agreement	0.21
T11 Right	71	53, 85	-0.01	-0.33, 0.32	0.41	0.10, 0.72	Moderate agreement	-0.01
T12 Right	85	69, 95	0.53	0.16, 0.89	0.71	0.47, 0.94	Substantial agreement	0.58
