# Supplementary material for: The interrater reliability of static palpation of the thoracic spine for eliciting tenderness and stiffness to test for a manipulable lesion
Source: Chiropr Man Therap. 2018 Dec 4;26:49. doi: 10.1186/s12998-018-0218-7 (PMC6278006; doi:10.1186/s12998-018-0218-7)
Supplement: Supplementary file 3 — Interexaminer reliability- Strict agreement- Pragmatic approach to assess segmental tenderness. Table of results for strict agreement for the pragmatic approach to assess segmental tenderness. (PDF 199 kb) [file 12998_2018_218_MOESM3_ESM.pdf]

Additional file 3: Interexaminer reliability- Strict agreement- Pragmatic approach to assess segmental tenderness

Spinal level	% Agreement	95% CI	Kappa	95%CI	PABAK	95% CI	Indicates	Kappa max
T1 Left	83	66, 93	0.63	0.36, 0.89	0.66	0.41, 0.91	Substantial agreement	0.83
T2 Left	86	70, 95	0.72	0.49, 0.94	0.71	0.48, 0.95	Substantial agreement	0.86
T3 Left	63	45, 79	0.28	0.01, 0.55	0.26	-0.06, 0.58	Fair agreement	0.50
T4 Left	63	45, 79	0.32	0.07, 0.56	0.26	-0.06, 0.58	Fair agreement	0.75
T5 Left	74	57, 88	0.51	0.27, 0.75	0.49	0.20, 0.77	Moderate agreement	1.00
T6 Left	71	54, 85	0.46	0.22, 0.70	0.43	0.13, 0.73	Moderate agreement	1.00
T7 Left	77	60, 90	0.55	0.28, 0.82	0.54	0.26, 0.82	Moderate agreement	0.71
T8 Left	80	63, 92	0.60	0.36, 0.86	0.60	0.33, 0.86	Moderate agreement	0.84
T9 Left	60	42, 76	0.26	0.00, 0.52	0.20	-0.12, 0.52	Slight agreement	0.55
T10 Left	69	51, 83	0.41	0.16, 0.66	0.37	0.06, 0.68	Fair agreement	0.79
T11 Left	71	54, 85	0.45	0.19, 0.71	0.43	0.13, 0.73	Moderate agreement	0.80
T12 Left	74	57, 88	0.48	0.19, 0.77	0.49	0.20, 0.77	Moderate agreement	0.51
T1 Right	80	63, 92	0.58	0.31, 0.85	0.60	0.33, 0.86	Moderate agreement	0.71
T2 Right	74	57, 88	0.46	0.16, 0.76	0.49	0.20, 0.77	Moderate agreement	0.49
T3 Right	63	45, 79	0.22	-0.11, 0.55	0.26	-0.06, 0.58	Fair agreement	0.23
T4 Right	74	57, 88	0.43	0.13, 0.73	0.48	0.20, 0.78	Moderate agreement	0.62
T5 Right	83	66, 93	0.60	0.32, 0.89	0.66	0.41, 0.91	Substantial agreement	0.69
T6 Right	80	63, 92	0.49	0.17, 0.82	0.60	0.33, 0.86	Moderate agreement	0.53
T7 Right	71	54, 85	0.34	0.01, 0.67	0.43	0.13, 0.73	Moderate agreement	0.39
T8 Right	80	63, 92	0.55	0.25, 0.84	0.60	0.33, 0.86	Moderate agreement	0.58
T9 Right	71	54, 85	0.43	0.14, 0.72	0.43	0.13, 0.73	Moderate agreement	0.56
T10 Right	77	60, 90	0.53	0.25, 0.81	0.54	0.26, 0.82	Moderate agreement	0.69
T11 Right	86	70, 95	0.70	0.46, 0.94	0.71	0.48, 0.95	Substantial agreement	0.85
T12 Right	89	73, 97	0.77	0.55, 0.98	0.77	0.56, 0.98	Substantial agreement	0.87
