# Supplementary material for: The interrater reliability of static palpation of the thoracic spine for eliciting tenderness and stiffness to test for a manipulable lesion
Source: Chiropr Man Therap. 2018 Dec 4;26:49. doi: 10.1186/s12998-018-0218-7 (PMC6278006; doi:10.1186/s12998-018-0218-7)
Supplement: Supplementary file 4 — Interexaminer reliability- Strict agreement- Standardized approach to assess segmental tenderness. Table of results for strict agreement for the standardized approach to assess segmental tenderness. (PDF 200 kb) [file 12998_2018_218_MOESM4_ESM.pdf]

Additional file 4: Interexaminer reliability- Strict agreement- Standardized approach to assess segmental tenderness

Spinal level	% Agreement	95% CI	Kappa	95%CI	PABAK	95% CI	Indicates	Kappa max
T1 Left	88	73, 97	0.70	0.44, 0.96	0.76	0.55, 0.98	Substantial agreement	1.00
T2 Left	68	49, 83	0.32	0.00, 0.65	0.35	0.04, 0.67	Fair agreement	0.35
T3 Left	68	49, 83	0.34	0.03, 0.66	0.35	0.04, 0.67	Fair agreement	0.42
T4 Left	76	59, 89	0.52	0.25, 0.79	0.53	0.24, 0.81	Moderate agreement	0.81
T5 Left	62	44, 78	0.25	-0.06, 0.56	0.23	-0.09, 0.56	Fair agreement	0.35
T6 Left	79	62, 91	0.58	0.32, 0.84	0.59	0.32, 0.86	Moderate agreement	0.83
T7 Left	74	56, 87	0.43	0.12, 0.74	0.47	0.17, 0.77	Moderate agreement	0.46
T8 Left	76	59, 89	0.51	0.22, 0.80	0.53	0.24, 0.81	Moderate agreement	0.67
T9 Left	79	62, 91	0.57	0.29, 0.85	0.59	0.32, 0.86	Moderate agreement	0.61
T10 Left	68	49, 83	0.28	-0.06, 0.61	0.35	0.04, 0.67	Fair agreement	0.30
T11 Left	79	62, 91	0.49	0.18, 0.81	0.59	0.32, 0.86	Moderate agreement	0.63
T12 Left	76	59, 89	0.44	0.13, 0.76	0.53	0.24, 0.81	Moderate agreement	0.61
T1 Right	82	65, 93	0.61	0.35, 0.87	0.65	0.39, 0.90	substantial agreement	1.00
T2 Right	65	46, 80	0.35	0.10, 0.60	0.29	-0.03, 0.61	Fair agreement	0.76
T3 Right	76	59, 89	0.50	0.21, 0.80	0.53	0.24, 0.81	Moderate agreement	0.58
T4 Right	62	44, 78	0.25	-0.04, 0.55	0.24	-0.09, 0.56	Fair agreement	0.42
T5 Right	71	53, 85	0.41	0.13, 0.70	0.41	0.10, 0.72	Moderate agreement	0.64
T6 Right	62	44, 78	0.31	0.06, 0.55	0.23	-0.09, 0.56	Fair agreement	0.74
T7 Right	65	46, 80	0.35	0.10, 0.60	0.29	-0.03, 0.61	Fair agreement	0.76
T8 Right	68	49, 83	0.39	0.14, 0.65	0.35	0.04, 0.67	Fair agreement	0.78
T9 Right	68	49, 83	0.38	0.12, 0.64	0.35	0.04, 0.67	Fair agreement	0.81
T10 Right	68	49, 83	0.38	0.12, 0.64	0.35	0.04, 0.67	Fair agreement	0.78
T11 Right	85	69, 95	0.69	0.45, 0.93	0.71	0.47, 0.94	Substantial agreement	0.84
T12 Right	76	59, 89	0.53	0.28, 0.79	0.53	0.24, 0.81	Moderate agreement	1.00
